# Isolate-specific rat brain transcriptional responses to rat lungworm (*Angiostrongylus cantonensis*)

**DOI:** 10.1093/femspd/ftaf003

**Published:** 2025-02-19

**Authors:** Phoebe Rivory, Rogan Lee, Jan Šlapeta

**Affiliations:** Sydney School of Veterinary Science, Faculty of Science, The University of Sydney, NSW 2006, Australia; NSW Health Pathology, Centre for Infectious Diseases and Microbiology Lab Services, Level 3 ICPMR, Westmead Hospital, Westmead, NSW 2145, Australia; School of Biomedical Sciences, Faculty of Medicine and Health, The University of Sydney, NSW 2006, Australia; Sydney School of Veterinary Science, Faculty of Science, The University of Sydney, NSW 2006, Australia; Sydney Infectious Diseases Institute, The University of Sydney, NSW 2006, Australia

**Keywords:** RNA-seq, transcriptomics, immunity, haplotype, angiostrongyliasis, Australia

## Abstract

The rat lungworm (*Angiostrongylus cantonensis*) is an invasive parasite of rats that in accidental hosts, such as dogs and humans, causes eosinophilic meningitis. In Australia, only two distinct rat lungworm *cox*1 haplotypes have been detected in clinically affected dogs, with haplotype Ac13 implicated in most cases. Using locally sourced isolates, we enquired whether the brain migrating larvae elicit different host response in its natural host. We examined brain transcriptome, faecal shedding rates, and adult worm of *A. cantonensis* isolates representing two distinct *cox*1 haplotypes, SYD.1 and Ac13 (represented by isolate SYD.2), in experimentally infected Wistar rats. For SYD.1-infected rats, only one differentially expressed gene (DEG) was upregulated in the compared to controls. In contrast, the transcriptome of SYD.2-infected rats included 100 DEGs, with enrichment of functional terms related to immune response, neuroactivity, and signalling. Faecal shedding did not differ between SYD.1- and SYD.2-infected rats, but adult worm burdens were higher in the SYD.1 group. The increased immune response in SYD.2-infected rats provides evidence that there is strain specific virulence that is pronounced in its natural host. This study provides initial parasite-specific evidence explaining why clinically affected dogs are more frequently presented with *A. cantonensis* haplotype Ac13.

## Teaser

Brain-invading *Angiostrongylus cantonensis* infects various animals, including humans, with brain reactions varying based on the parasite’s specific identity. Two different Australian isolates of rat lungworm (*Angiostrongylus cantonensis*) trigger different responses from the definitive (rat) host during experimental infection.

## Introduction

The rat lungworm (*Angiostrongylus cantonensis*) actively migrates into the brains of its hosts as part of its normal life cycle and is the leading cause of eosinophilic meningitis in humans worldwide (Wang et al. 2008, Cowie 2013). The definitive *Rattus* spp. hosts rarely suffer clinical disease; however, infection of accidental hosts (e.g. humans, dogs) can result in a severe, and occasionally fatal, meningitic disease called neural angiostrongyliasis (Mason 1987, Alicata 1991, Martins et al. 2015). Mammalian hosts (both natural and accidental) become infected via the ingestion of infective larvae that develop inside intermediate molluscan (e.g. slug and snail) hosts (Bhaibulaya 1975, Alicata 1991).

In the rat, after ingestion, L3s (infective third-stage larvae) migrate to the central nervous system (CNS) and at 6 days post-infection, most of the larvae are within the brain (Bhaibulaya 1975). The larvae develop further before migrating to the pulmonary arteries, where adults mate and release eggs that hatch into first-stage larvae (L1s). The L1s then travel to the pharynx, are swallowed, and are excreted in faeces. In accidental (non-permissive) mammalian hosts, rat lungworm L3s cause pathology via larval migration through the CNS, but the intense immune response and eosinophil infiltration into the meninges is what drives clinical disease (Murphy and Johnson 2013, Martins et al. 2015, Barratt et al. 2016). Ultimately the rat lungworm larvae usually do not develop much further in these non-permissive hosts and may die, further triggering the immune system. Eosinophils secrete molecules such as interleukin (IL)-4, 5, 6, 10, 12, 13, 18, transforming growth factor (TGF)-α/β, leukotrienes, proteases, reactive oxygen species, and nitric oxide (NO), in an attempt to provide a protective immune response, but also contribute to host-induced pathophysiology (Behm and Ovington 2000, Gosnell and Kramer 2013, Zhou et al. 2022, Lee et al. 2023). On the other hand, the definitive rat host is capable of tolerating moderate doses of infective L3s, with doses exceeding 200 worms resulting in neurological impairment (Ji et al. 2017). Nevertheless, a complete understanding of the immune pathways that dictate disease manifestation in both definitive and non-permissive hosts is lacking.

In Australia, there are two *A. cantonensis cox*1 haplotypes known (Ac13 and SYD.1), with both causing clinical disease in dogs (Mallaiyaraj Mahalingam et al. 2021). The mortality rate of this disease, also known as canine neural angiostrongyliasis (CNA), is 14%–58% (Lunn et al. 2012), and diagnosis and treatment remain complicated, invasive, and expensive (Walker et al. 2015). Studies from our group have shown that the majority of clinical cases are associated with infection by the Ac13 haplotype; although, the total number of published CNA cases with confirmed *cox*1 haplotype determined is currently small (*N =* 15) (Mallaiyaraj Mahalingam et al. 2021, Baláž et al. 2023). Our investigations on pestiferous rats in a western Sydney zoo and in Sydney’s central business district (CBD) suggest that these haplotypes may have distinct geographical ranges. Brown rats (*Rattus norvegicus*) from the western Sydney zoo were primarily harbouring the Ac13 haplotype, whereas *R. norvegicus* and black rats (*Rattus rattus*) from the CBD were found to harbour the SYD.1 haplotype (Rivory et al. 2023, Rivory et al. 2024).

A previous study comparing two Brazilian rat lungworm haplotypes (ac8 and ac9) in definitive (rat) and non-permissive (mice) hosts revealed differences in pathogenicity, infectivity, and fecundity (Monte et al. 2014)—warranting inquiry into the two endemic Australian haplotypes. Although genotypic features of an *A. cantonensis* isolate or haplotype may not necessarily imply a level of pathogenicity, there appears to be genetic uniformity among *cox*1 haplotypes that have invaded geographical areas outside of the parasite’s original endemic location—including SYD.1, Ac13, and Brazilian haplotypes ac8 and ac9 (Červená et al. 2019). The culpability of the Ac13 haplotype in neural angiostrongyliasis cases of dogs and a squirrel monkey (Mallaiyaraj Mahalingam et al. 2021, Rivory et al. 2023) raises concerns that Ac13-type larvae may be more pathogenic. Alternatively, Ac13 may be causing CNA more often due to other factors, such as increased fecundity in the definitive host—which would enhance its survival and proliferation in rat and intermediate host populations (Monte et al. 2014).

Considering the differences in the distribution of Australian *A. cantonensis cox*1 haplotypes Ac13 and SYD.1, and the fact that a majority of (sequenced) accidental host cases have identified Ac13 as the culprit, this study aims to investigate biological differences between SYD.1 and Ac13 haplotypes in permissive hosts. To do so, we established a laboratory culture of a locally sourced isolate (‘SYD.2’) that matches the Ac13 haplotype on the partial *cox*1 region. We utilised experimental infection of Wistar rats and subsequent RNA sequencing of brain tissue at 6 days post-infection to assess if the host’s response to the neural migration of the parasite differs depending on the *A. cantonensis* isolate given (SYD.1 or SYD.2). Additionally, we measured the efficiency of parasite establishment and transmission by counting total adult worm burden and L1 shedding, respectively, in another cohort of rats. We expect to see that (1) SYD.2 triggers a more intense immune response—an indicator of higher pathogenicity; or (2) SYD.2-infected rats shed more L1s—which would improve transmission to the intermediate host. This research will clarify if there are any differences between the host–parasite interactions of *A. cantonensis* Ac13 and SYD.1 haplotypes exist and further contribute to the growing understanding of the immune response to Australian *A. cantonensis* isolates in definitive hosts.

## Materials and methods

### Ethics statement

All mammals involved in this study were approved for use by the relevant animal ethics committees. The use of Wistar rats for the production of infective larvae and for the experimental infection was approved by the Western Sydney Local Health District Animal Ethics Committee (approval numbers: 8003.03.18 and 4389.08.23, respectively).

### Preparation of *A. cantonensis* larvae

*Angiostrongylus cantonensis* SYD.1 and SYD.2 isolates used in this study were cultivated separately. The SYD.1 isolate originated from a wild rat (*Rattus norvegicus*) in Taronga Zoo, Sydney, Australia (Červená et al. 2019), and has been maintained at Westmead Hospital for over two decades by regular passage through Wistar rats and aquatic snails [*Bullastra lessoni*; culture methods described previously (Pai et al. 2022)]. Culture of SYD.2 was established in 2023 (typed as mtDNA haplotype Ac13) using the same methods as described above between Wistar rats and aquatic snails (*B. lessoni*), where the larvae were originally sourced from opportunistically collected rat faeces at Sydney Zoo, Sydney, Australia. Confirmation of haplotype was performed by partial *cox*1 real-time (rt)-PCR and sequencing of DNA from L1s and L3s prior to and after the first passage using methods described previously (Rivory et al. 2023).

To produce third-stage larvae (L3s) for the experimental infection, first-stage larvae (L1s) were collected from infected rat faeces via the Baermann technique (Mackerras and Sandars 1955). Then, L1s were washed via two rounds of centrifugation (400 × *g* for 10 min) and resuspension with reverse-osmosis (RO) water (Pai et al. 2022, Rivory et al. 2023a, 2023b). *Bullastra lessoni* snails were exposed to either SYD.1 or SYD.2 L1s, at a rate of ∼500 larvae per snail, in a large glass dish topped up with RO water for 6 h. Snails were housed (separately according to isolate given) for at least a month in 25°C and 80% humidity aquarium tanks fitted with an air pump and crushed marble substrate. The snails were fed rinsed lettuce *ad libitum*, and tank water was replaced as necessary. One day prior to experimental infection, snails with their shells removed were quickly crushed and placed in a Petri dish with RO water to release the infective L3s from the snail tissues. L3s were stored in 0.5 ml aliquots of 30 at room temperature on the day of experimental infection/s.

### Comparative RNA-seq experiment

#### Experimental infection of Wistar rats

After 2 weeks of acclimation in the animal house, 13-week-old male Wistar rats (*N =* 18; Ozgene, Australia) were randomly allocated into three treatment groups of six: (1) SYD.1, (2) SYD.2, and (3) mock control. The weight (g) of individual rats was recorded, and a one-way ANOVA with Tukey’s multiple comparisons was performed to confirm the weights were equally distributed between groups. Rats were lightly sedated with isoflurane gas, then a blinded researcher gavaged each rat with 0.5 ml water containing 30x SYD.1 L3s (SYD.1 *cox*1 haplotype), 30x SYD.2 L3s (Ac13 *cox*1 haplotype), or no larvae (mock control) according to their allocated treatment groups. Rats were returned to their housing in pairs—one of each pair was marked on the tail to identify individuals. Commercial rat pellets and water were provided *ad libitum*, and wood shavings and cardboard tubes provided for enrichment. At 6-days post-infection, when the larvae have migrated to the brain and moulted once (Bhaibulaya 1975), the rats were euthanized via gaseous CO_2_. The whole brain was removed, and an ∼2 mm wide coronal section of cerebral tissue was dissected from each rat and homogenized on a Petri dish with a sterile scalpel blade. This tissue section was chosen as *A. cantonensis* larvae tend to aggregate on the cerebral surface of infected rats (Bhaibulaya 1975, OuYang et al. 2012). Aliquots of homogenized tissue (∼20 mg) were placed in cryovials and flash frozen in liquid nitrogen vapour, temporarily kept on dry ice, and finally stored at −80°C.

#### Molecular detection of larvae presence in rat brain tissue

To detect the presence of *A. cantonensis* larvae in the brain tissues of experimentally infected rats, genomic DNA was isolated from two of the remaining homogenized brain tissue frozen aliquots from each of the 18 samples (total *N =* 36). Genomic DNA was isolated using the Monarch Genomic DNA Purification Kit (tissue protocol; New England Biolabs, Australia) according to manufacturer’s instructions and eluted twice with a final volume of 60 µl.

Firstly, to confirm successful DNA isolation, partial ß-actin was amplified with primers ActinF2 (ACC ACT GGT ATT GTC ATG GAC TCT G) and ActinR2 (GCT CTT CTC CAG GGA GGA CGA) (Rishniw et al. 2006). Primers were first checked for suitability *in silico* against *Rattus norvegicus* ß-actin (Actb; NCBI reference sequence: NM_031144.3). Reactions (20 µl) included 10 µl of SensiFAST SYBR No-ROX Kit (Bioline, Australia), each primer at 0.4 µM concentrations, PCR-grade water, and 2 µl of DNA template. The rt-PCR cycling conditions began with a denaturation step at 95°C for 3 min, followed by 40 two-step cycles at 95°C for 5 s and 60°C for 15 s. The rt-PCR was carried out on a CFX96 Touch rt-PCR Detection System with the corresponding CFX Manager v.3.1 software (Bio-Rad, Australia). Each run included a positive control (rat skin DNA), a ddH_2_O no-template control, and a blank extraction control. The threshold was determined automatically via default settings, and samples with cycle threshold (Ct) values <30 were considered positive. All samples (*N =* 36) passed this QC step.

To detect ‘active infection’ (or, the presence) of *A. cantonensis* larvae in the brain tissues of experimentally infected rats, each sample was subjected to a hypersensitive probe-based rt-PCR assay that targets a repeat contig (AcanR3990) (Sears et al. 2021). Reactions were run at a final volume of 20 µl, including 2 µl of gDNA and 10 µl of Luna Universal Probe qPCR Master Mix (New England BioLabs, Australia). Final concentrations of primers and probe were 0.4 and 0.1 µM, respectively (Integrated DNA Technologies, Inc., Australia). The rt-PCR run included an extraction control, ddH_2_O no-template control, and positive control (0.1 × *A. cantonensis* L1 DNA). Reactions were performed in a CFX96 Touch rt-PCR Detection System (Bio-Rad Laboratories, Inc.) with the following cycling conditions: 95°C for 3 min; and 40 cycles of 95°C for 5 s, and 60°C for 15 s. Amplification curves and Ct values were recorded using CFX Maestro Software 2.3 (Bio-Rad Laboratories, Inc.). The threshold was determined automatically via default settings. Samples with Ct-values < 35 in at least one aliquot were considered positive.

#### RNA isolation, sequencing, and mapping

Total RNA was isolated from 10 mg of frozen homogenized brain tissue using the Monarch® Total RNA Miniprep Kit (New England BioLabs, Australia). RNA was subsequently lyophilized and stored in Novogene RNA Ambient Tubes according to manufacturer’s instructions for shipping to Novogene, Singapore. Directional mRNA library preparation (polyA enrichment), QC, mapping and read quantification were performed by Novogene. Briefly, sequencing was conducted on an Illumina NovaSeq platform, aiming for 9G of raw data per sample with paired-end 150 bp reads. Resulting sequencing reads were filtered to obtain clean reads by removing reads with adapter contamination, reads with > 10% of uncertain (N) nucleotides, and reads when low-quality nucleotides (base quality < 5) constitute more than 50% of the read. Filtered RNA-seq reads were aligned to the reference genome (*Rattus norvegicus* mRatBN7.2) using HISAT2 [version 2.0.5; https://daehwankimlab.github.io/hisat2 (Kim et al. 2015)] with the following parameters: -p 4 –dta -t –phred33. The aligned reads were then sorted into BAM files using SAMtools [version 1.4.1; https://www.htslib.org (Danecek et al. 2021)], and featureCounts [version 1.5.0-p3; https://subread.sourceforge.net/featureCounts.html (Liao et al. 2014)] was used to count the reads mapped to genomic features. The annotation file used was a GTF file, and the following parameters were applied: -t exon -g gene_id. Default parameters of featureCounts were used, and no filtering was applied to exclude transcripts with the class code ‘u’ (unknown, intergenic transcripts). As a result, all transcripts, including those classified as ‘u’, were included in the read counts.

#### Differentially expressed gene identification and functional analyses (KEGG and GO)

Principal component analysis (PCA), differential expression analysis, the creation of hierarchical-clustered heatmaps, and follow-up functional analyses were all carried out on the cloud platform NovoMagic (Novogene, Singapore, https://magic.novogene.com/). Principal components were calculated using FKPM values. Figures (excluding heatmaps) were produced in GraphPad Prism (Version 9 for Windows, USA). Differentially expressed genes (DEGs) were determined using the DESeq2 (v1.20.0) R package (Love et al. 2014), with adjusted *P*-values (*P*_adj_) being calculated using Benjamini and Hochberg’s approach for controlling the false discovery rate. DEGs were considered significant if their *P*_adj_ < 0.05 and |log_2_(fold change)| > 2. Note that analyses were repeated with less stringency [*P*_adj_ < 0.05 and |log_2_(fold change)| > 1] due to low signal. Heatmaps with hierarchical clustering of genes by (1) top 200 DEGs sorted by smallest *p*_adj_ and (2) significant DEGs only were generated from the log_2_(FPKM + 1) values of genes, and row scores were normalized (‘*Z*-scores’). Gene ontology (GO) enrichment and Kyoto Encyclopedia of Genes and Genomes (KEGG) pathway analysis were based on all DEGs from each comparison. KEGG and GO terms with *P*_adj_ < 0.05 were considered significantly enriched. The top 10 significant KEGG and GO terms were visualized with bubble plots and bar plots, respectively.

Using the protocol above, a total of seven different comparisons were investigated. Comparison groups were (1) marked control rats vs. unmarked control rats to identify noise within the untreated group; (2) SYD.1-infected rats vs. control rats; (3) SYD.2-infected rats vs. control rats; (4) AcanR3990 PCR -positive (‘actively’ infected) vs. AcanR3990 PCR-negative rats; (5) AcanR3990 PCR-positive (‘actively’ infected) vs. control rats; (6) treated rats (= pooled SYD.1- and SYD.2-infected) vs. control rats; and (7) SYD.1-infected vs. SYD.2-infected rats. Initially, the data were explored visually using PCA plots, volcano plots, and clustered heatmaps to see trends in the data across these seven comparison groups. Comparisons were further explored using less stringent DEG selection to account for low signal and allow more genes to be used to inform subsequent functional analysis. For each analysis, KEGG and GO plots were produced (if there were any significantly enriched pathways/terms) for interpretation of the data in a biological context.

### Total adult worm burden and L1 shedding

To track the shedding of L1s in the faeces of rats infected with SYD.1 and SYD.2 isolates, four Wistar rats (two per isolate) were infected with *A. cantonensis* using the same procedures described above. At 6-, 8-, and 10-weeks post-infection, the faeces expelled by the rats over a 24-h period was collected and immediately allowed to soften with RO water. The faeces was mixed into a slurry and placed in a Baermann apparatus for 24 h. The sediment was collected and washed via centrifugation at 400 × *g* for 10 min before resuspension to 10 ml. Three 20 µl aliquots were taken from each vortexed suspension to count L1s under a light microscope. The average of the three counts was taken to estimate L1s per gram of faeces (L1s/g), which was then plotted in GraphPad Prism (Version 9 for Windows, USA). A Mann–Whitney test with multiple comparisons was performed to statistically evaluate differences in shedding between SYD.2 and SYD.1 groups at each timepoint. As these rats were used for maintaining laboratory isolates of *A. cantonenis*, a total (adult) worm count was performed when they were eventually euthanized at 38 weeks post-infection. Rats were euthanized via CO_2_, then the cardio-respiratory organs were removed. Worms were retrieved by gently teasing apart lung and heart tissues with fine tweezers, and adult worms were sexed and counted. Total worm count means (± SD) for each group were plotted, and a *t*-test was performed; and sex ratios were calculated.

## Results

As expected, due to the dose of L3s given intended to cause a non-clinical level of disease, there were no mortalities or treatment-related adverse reactions over the course of the study.

### Detection of *A. cantonensis* larvae in experimentally infected rats via AcanR3990 rt-PCR

All samples (*N =* 36) passed the first QC step testing for sufficient gDNA isolation via β-actin rtPCR. For the AcanR3990 rt-PCR, both aliquots from all control rats were negative. Five out of 12 *A. cantonensis*-infected rat brain samples amplified with Ct-values < 35 in at least one of the two aliquots, deeming them positive for *A. cantonensis* larvae in the brain. Of these five positive samples, two were from rats infected with the SYD.1 isolate (4U_S and 7U_S) and three were from rats infected with the SYD.2 isolates (1M_A, 3M_A, and 3U_A); resulting in 33.3% and 50% ‘active’ infection rates, respectively. A summary of the β-actin and AcanR3990 PCRs is detailed in Supplementary Table 2.

### RNA-sequencing and mapping statistics

Sequencing generated a total of 1201888726 raw reads, averaging 66769271.33 ± SD 9073296.94 reads per sample. Detailed sequencing metrics, including base quality scores, GC content, and mapping efficiency, are summarized in Supplementary Table 1. Overall, high-quality reads were obtained, with average base quality scores of Q20 = 98.11 ± SD 0.08 and Q30 = 94.87 ± SD 0.18. An average of 97.05% ± SD 0.18% of reads were successfully mapped to the *Rattus norvegicus* reference genome (mRatBN7.2).

### Differential gene expression and functional analyses

**Figure 1.**
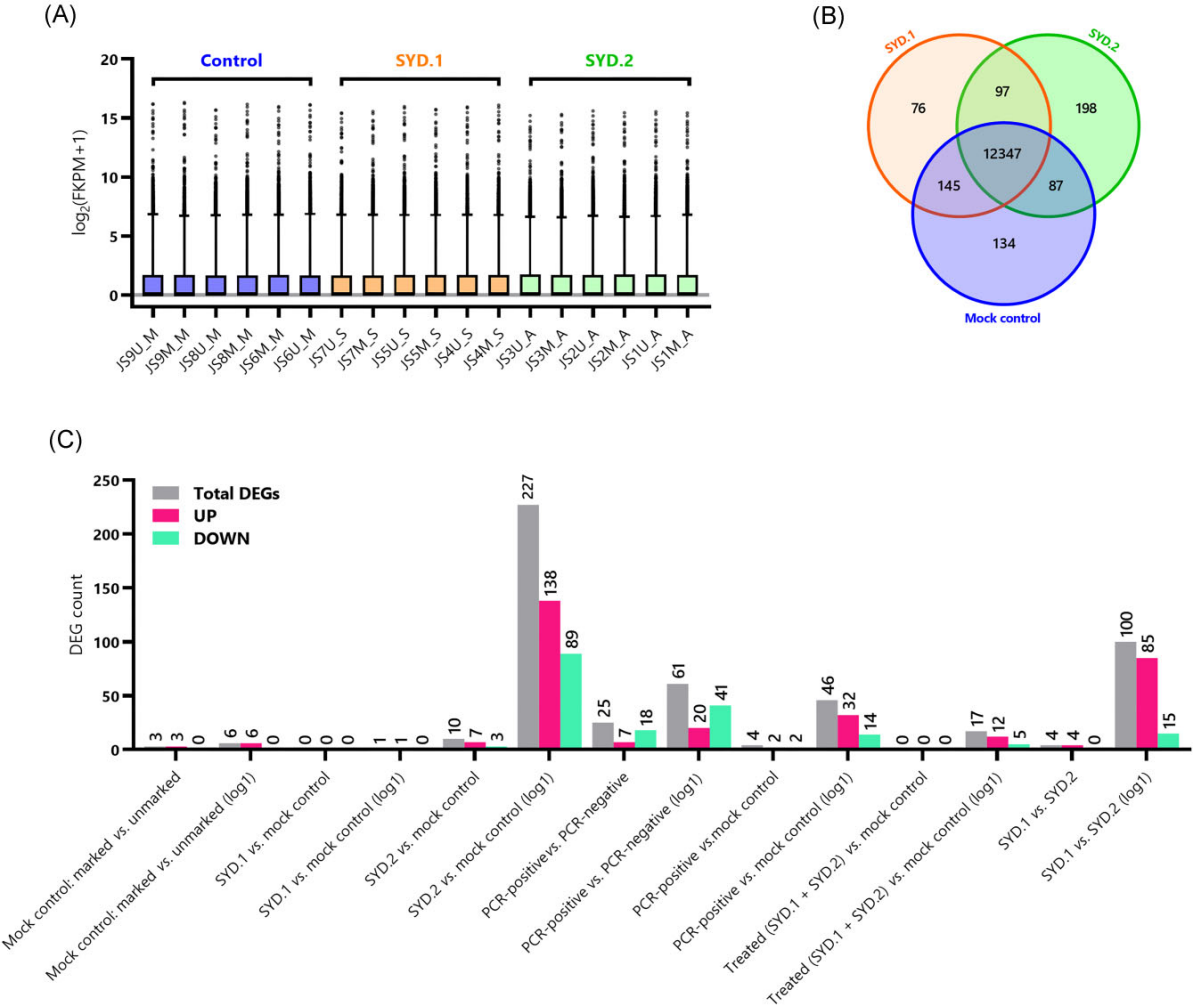
Overview of gene expression amongst samples and results from differential gene expression analysis of Wistar rats experimentally infected with *A. cantonensis* isolates, SYD.1 and SYD.2, and uninfected controls. (A) Boxplot of gene read distribution for all rat brain samples [as log_2_(FKPM + 1); *N =* 18] by treatment group: mock control (blue), *A. cantonensis* SYD.1-infected (orange), and *A. cantonensis* SYD.2-infected (green). Boxes indicate the central 50% of values, and whiskers represent the 1–99th percentiles. (B) Venn diagram depicting shared and unique DEGs in each of the three groups (SYD.1, SYD.2 and mock control). (C) Summary of all DEG analyses for seven comparisons with selection criteria |log_2_(fold change)| > 2 and |log_2_(fold change)| > 1 [given the suffix ‘(log1)’]. Grey bars indicate the total number of DEGs discovered in each comparison, with the number of upregulated (pink) and downregulated (light green) genes also shown.

Gene read distribution was consistent across all samples (Fig. 1A). Overall, differential gene expression and functional analyses were undertaken on a total of seven different comparisons. The samples included in each comparison group, the parameters, total number of transcripts, number of upregulated and downregulated DEGs, significant KEGG pathways, and significant GO terms are displayed in Table 1. A core transcriptome of 12347 genes was shared among SYD.1, SYD.2, and mock control groups; with 97 shared between SYD.2 and SYD.1, 145 shared between mock control and SYD.1, and 87 shared between SYD.2 and mock control (Fig. 1B). The SYD.2 vs. mock control comparison identified the most DEGs [|log_2_(fold change)| > 1; 227 DEGs], followed by SYD.2 vs. SYD.1 [|log_2_(fold change)| > 1; 100 DEGs], indicating a more pronounced transcriptional response to the *A. cantonensis* SYD.2 isolate (Fig. 1C).

**Table 1.** Results from differential expression (DEG) and functional (KEGG and GO) analyses.

#	Analysis name	Group 1 samples	Group 2 samples	Parameters*	Total transcripts	Up DEGs	Down DEGs	Significant* KEGGs/total	Significant* GOs/total
1	Mock control: marked vs. unmarked	JS6M_M, JS8M_M and JS9M_M	JS6U_M, JS8U_M and JS9U_M	α = 0.05, |log_2_(FC)| > 2	24181	3	0	5/5	42/43
1.1				α = 0.05, |log_2_(FC)| > 1		6	0	0/8	84/279
2	SYD.1 vs. mock control	JS4M_S, JS4U_S, JS5M_S, JS5U_S, JS7M_S and JS7U_S	JS6M_M, JS8M_M, JS9M_M, JS6U_M, JS8U_M and JS9U_M	α = 0.05, |log_2_(FC)| > 2	25 887	0	0	N/A	
2.1				α = 0.05, |log_2_(FC)| > 1		1	0	23/23	257/257
3	SYD.2 vs. mock control	JS1M_A, JS1U_A, JS2M_A, JS2U_A, JS3M_A and JS3U_A	JS6M_M, JS8M_M, JS9M_M, JS6U_M, JS8U_M and JS9U_M	α = 0.05, |log_2_(FC)| > 2	25 912	7	3	1/7	22/225
3.1				α = 0.05, |log_2_(FC)| > 1		138	89	10/175	120/2 981
4	Treated: PCR-positive vs. PCR-negative	JS1M_A, JS3M_A, JS3U_A, JS4U_S and JS7U_S	JS1U_A, JS2M_A, JS2U_A, JS4M_S, JS5M_S, JS5U_S and JS7M_S	α = 0.05, |log_2_(FC)| > 2	26 048	7	18	2/32	11/735
4.1				α = 0.05, |log_2_(FC)| > 1		20	41	1/58	22/1 662
5	PCR-positive vs. mock control	JS1M_A, JS3M_A, JS3U_A, JS4U_S and JS7U_S	JS6M_M, JS8M_M, JS9M_M, JS6U_M, JS8U_M and JS9U_M	α = 0.05, |log_2_(FC)| > 2	25 645	2	2	5/5	95/225
5.1				α = 0.05, |log_2_(FC)| > 1		32	14	6/57	58/1 188
6	Treated (SYD.1 + SYD.2) vs. mock control	JS1M_A, JS1U_A, JS2M_A, JS2U_A, JS3M_A, JS3U_A, JS4M_S, JS4U_S, JS5M_S, JS5U_S, JS7M_S and JS7U_S	JS6M_M, JS8M_M, JS9M_M, JS6U_M, JS8U_M and JS9U_M	α = 0.05, |log_2_(FC)| > 2	26 839	0	0	N/A	
6.1				α = 0.05, |log_2_(FC)| > 1		12	5	0/28	0/406
7	SYD.2 vs. SYD.1	JS1M_A, JS1U_A, JS2M_A, JS2U_A, JS3M_A and JS3U_A	JS4M_S, JS4U_S, JS5M_S, JS5U_S, JS7M_S and JS7U_S	α = 0.05, |log_2_(FC)| > 2	26 048	4	0	47/50	75/91
7.1				α = 0.05, |log_2_(FC)| > 1		85	15	1/150	29/2 158

FC = fold change; DEG = differentially expressed genes, KEGG = Kyoto Encyclopedia Kyoto Encyclopedia of Genes and Genomes, GO = gene ontology. *DEG analysis was performed using DESeq2 and adjusted *P*-values were used to determine statistical significance for DEGs, and GO and KEGG terms (α = 0.05).

#### Testing for noise: marked control *vs*. unmarked control rats

PCA plot of the marked control rats (JS6M_M, JS8M_M, and JS9M_M) vs. unmarked control rats (JS6U_M, JS8U_M, and JS9U_M) revealed some grouping according to cage number (Supplementary Fig. 1A). The clustered heatmap reveals a degree of heterogeneity within groups, and there is a little indication of group-specific expression patterns (Supplementary Fig. 1B). Differential expression analysis revealed a total of 24 181 unique transcripts, with three DEGs (Prr32, Slco1a5, and Cldn2) being upregulated in the marked group (Table 1; Supplementary Fig. 1C). These three genes were not observed in the DEG lists from any subsequent analyses. Using less stringent DEG selection criteria [*P*_adj_ < 0.05 and |log_2_(fold change)| > 1], the number of DEGs rose to only six (Table 1).

#### *Angiostrongylus cantonensis* SYD.1-infected rats expression profile was virtually no different to control rats

**Figure 2.**
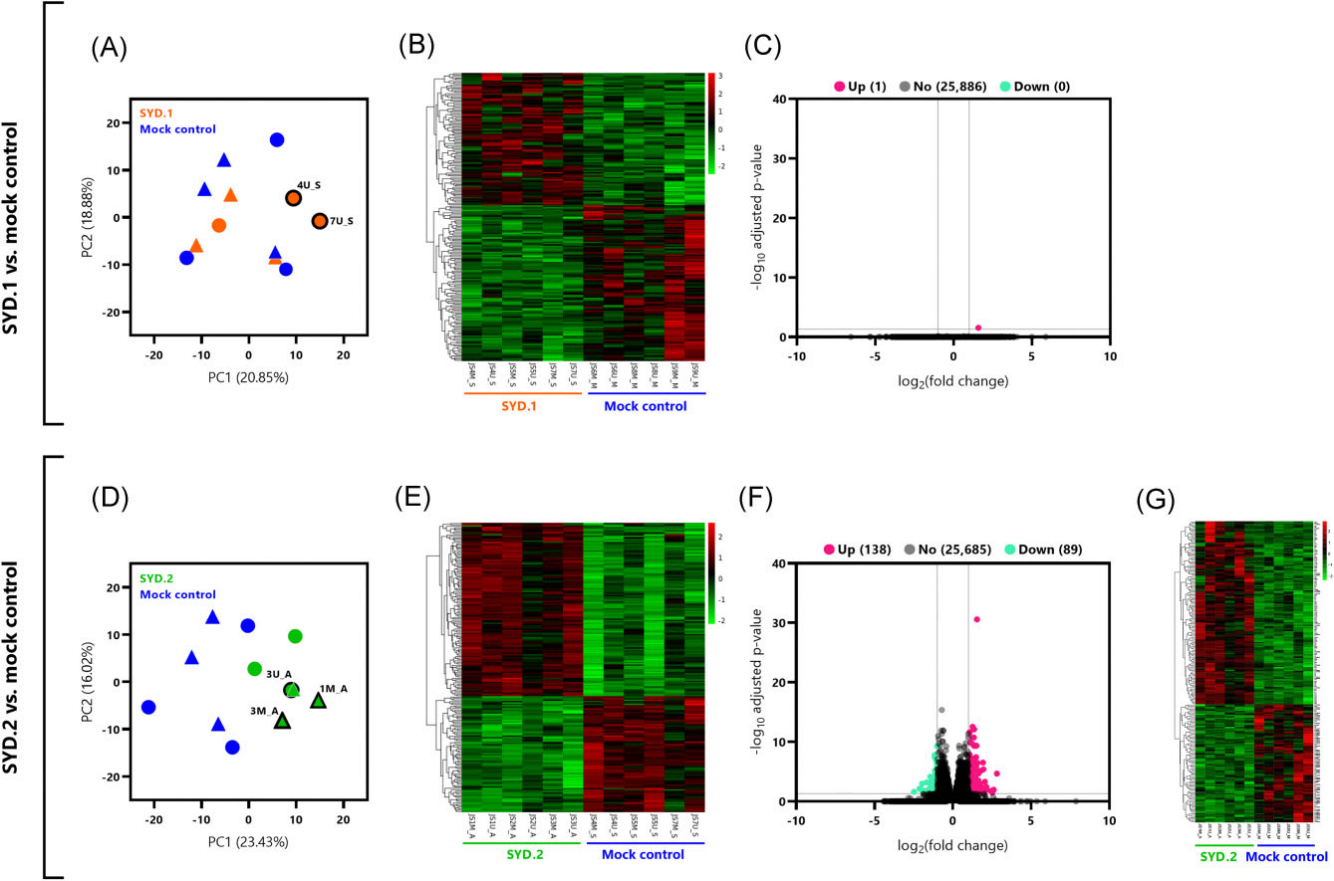
Overview of differential gene expression analysis comparing rats infected with *A. cantonensis* isolates SYD.1 and SYD.2 to uninfected rats (mock controls). Plots (A–C) are from the SYD.1 vs. mock control comparison, and plots (D–F) are from the SYD.2 vs. mock control comparison. (A) Plot from PCA of FKPM values from SYD.1 (orange) and mock control (blue) groups. Marked rats are indicated by triangles and unmarked by circles. Two AcanR3990-rtPCR-positive samples are labelled and outlined in black. (B) Hierarchical clustered heatmap of the top 200 genes (by smallest *P*_adj_) between SYD.1 and mock control groups. Gene expression levels [as log_2_(FPKM + 1)] were normalized by row (*Z*-scores), which are indicated by red-green colour. (C) Volcano plot depicting DEGs identified by comparing SYD.1 and mock control groups, with upregulated genes in pink, downregulated genes in green, and non-significant genes in black. DEGs were selected based on |log_2_(fold change)| > 1 and adjusted *P*_adj_ < 0.05. The log_2_(fold change) values are on the *x*-axis, and *P*_adj_ values (transformed by −log_10_) are displayed on the *y*-axis. (D) PCA plot, generated as above, but for the SYD.2 (green) vs. mock control (blue) comparison. Three samples were AcanR3990-rtPCR-positive, as indicated by labels and a thick black outline. (E) Hierarchical clustered heatmap of top 200 genes, generated as above, but for the SYD.2 vs. mock control comparison. (F) Volcano plot, generated as above, but for the SYD.2 vs. mock control comparison. Note the much stronger signal (i.e. number of differentially expressed genes; DEGs) in this analysis compared to the above SYD.1 vs. mock control. (G) Hierarchical clustered heatmap of the 227 DEGs found in the SYD.2 vs. mock control comparison. Gene expression levels [as log_2_(FPKM + 1)] were normalized by row (*Z*-scores), which are also indicated by red-green colour gradient.

To determine the host response to infection with *A. cantonensis* SYD.1, the SYD.1-infected rats (JS4M_S, JS4U_S, JS5M_S, JS5U_S, JS7M_S, and JS7U_S) were compared to the control group (JS6M_M, JS8M_M, JS9M_M, JS6U_M, JS8U_M, and JS9U_M). In the PCA plot and clustered heatmap of top 200 genes (by smallest *P*_adj_), it appears that there is limited clustering of these groups (Fig. 2A and B). Notably, the two SYD.1-infected rats with positive AcanR3990 PCR results (JS4U_S and JS7U_S) were slightly separated from the rest of the dataset. Differential gene expression analysis revealed no DEGs out of a total of 25 887 transcripts (Table 1). When selection criteria were relaxed to |log_2_(fold change)| > 1, only one gene was found to be differentially expressed (RT1-Db1, *P*_adj_ = 0.027; Fig. 2C).

#### Genes differentially expressed in *A. cantonensis* SYD.2-infected rats

**Figure 3.**
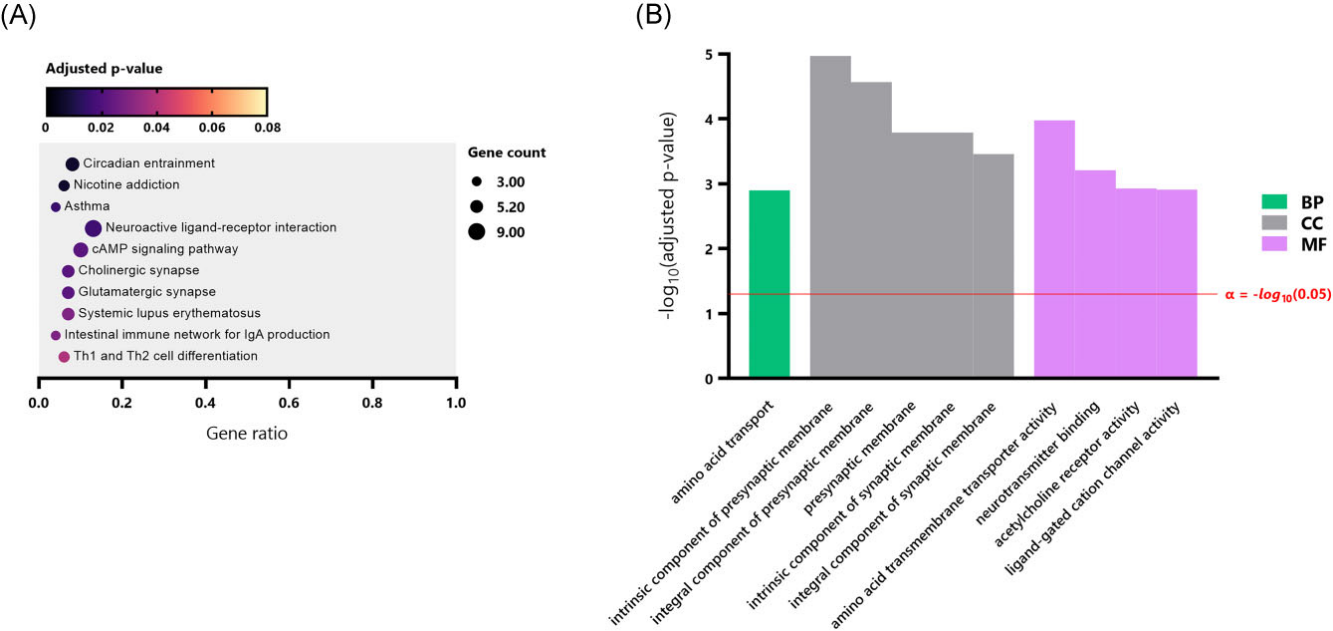
Subsequent functional enrichment analysis of the DEGs (*N =* 227) identified between rats infected with *A. cantonensis* SYD.2 isolate (=Ac13 *cox*1 haplotype) and uninfected controls. (A) Bubble plot depicting the top 10 significant KEGG (Kyoto Encyclopedia of Genes and Genomes) pathways. Significance was determined by *P*_adj_ < 0.05. Bubble size represents the number of DEGs within the pathway, colour indicates the *P*_adj_ value, and the gene ratio (number of DEGs/total genes in the pathway) is displayed on the *x*-axis. (B) Bar plot of the top 10 significant GO terms. Significance was determined by *P*_adj_ < 0.05, and selected by the smallest *P*_adj_. *P*_adj_ values (−log_10_ transformed) are shown on the *y*-axis, and GO terms are on the *x*-axis. Green bars represent biological processes (BP), grey bars represent CC, and pink bars represent MF. The red line indicates the significance threshold (α = 0.05).

To investigate the host response to infection with the *A. cantonensis* SYD.2 isolate, the SYD.2-infected rats (JS1M_A, JS1U_A, JS2M_A, JS2U_A, JS3M_A, and JS3U_A) were compared to the control group (JS6M_M, JS8M_M, JS9M_M, JS6U_M, JS8U_M, and JS9U_M). In the PCA plot and clustered heatmap of top 200 genes (by smallest *P*_adj_), distinct separation of the two groups is visible (Fig. 2D and E). The three AcanR3990 PCR-positive samples (S1M_A, JS3M_A, and JS3U_A) appeared to cluster together in the PCA (Fig. 2D). Differential gene expression analysis of a total of 25 912 discovered transcripts revealed 10 DEGs; seven being upregulated and three being downregulated (Table 1). Initial KEGG and GO enrichment analyses were unsuccessful due to a small number of DEGs. After reanalysis with more relaxed DEG selection criteria [|log_2_(fold change)| > 1], a total of 227 DEGs were identified, with 138 being upregulated and 89 being downregulated (Fig. 2F). The clustered heatmap of these DEGs shows substantial separation of the SYD.2 and control groups (Fig. 2G). Subsequent KEGG pathway enrichment analysis of the 227 DEGs produced a total of 175 pathways, with 10 of those being significant (Table 1). In order of smallest *P*_adj_, the KEGG pathways enriched were ‘circadian entertainment’, ‘nicotine addiction’, ‘asthma’, ‘neuroactive ligand-receptor interaction’, ‘cAMP signalling pathway’, ‘cholinergic synapse’, ‘glutamatergic synapse’, ‘systemic lupus erythematosus’, ‘intestinal immune network for IgA production’, and ‘Th1 and Th2 cell differentiation’ (Fig. 3A). Out of 2981 GO terms, 120 were significant (Table 1). The top 10 GO terms (by smallest *P*_adj_) included one biological process (BP; ‘amino acid transport’), five cellular components (CC; all were terms regarding the ‘synaptic membrane’), and four molecular functions (MF; ‘amino acid transmembrane transporter activity’, ‘neurotransmitter binding’, ‘acetylcholine receptor activity,’ and ‘ligand-gated cation channel activity’) (Fig. 3B).

#### ‘Actively’ infected (PCR-positive) rats associated with synapses and ligand-receptor interactions

Another two sets of DEG and functional analyses were performed in an attempt to further understand the apparent discrimination from AcanR3990 rt-PCR-positive samples (JS1M_A, JS3M_A, JS3U_A, JS4U_S, and JS7U_S) from their PCR-negative (JS1U_A, JS2M_A, JS2U_A, JS4M_S, JS5M_S, JS5U_S, and JS7M_S) and uninfected (mock control) counterparts (JS6M_M, JS8M_M, JS9M_M, JS6U_M, JS8U_M, and JS9U_M). PCA plots revealed that samples were well separated for both the PCR-positive vs. PCR-negative and PCR-positive vs. control analyses (Supplementary Fig. 2A and D, respectively). The top 200 genes (by smallest *P*_adj_) in clustered heatmaps also reveal a distinction between groups (Supplementary Fig. 2B and E, respectively). The initial DEG analysis returned a total of 26 048 and 25 645 transcripts, respectively (Table 1). DEG analysis of PCR-positive vs. PCR-negative, gave a total of 25 DEGs, with seven being upregulated and 18 being downregulated. Upon reanalysis with reduced stringency [|log_2_(fold change)| > 1], the number of DEGs increased to 61 (20 upregulated, and 41 downregulated; Supplementary Fig. 2C). Similarly, for the PCR-positive vs. mock control comparison, four DEGs (two upregulated and two downregulated) were identified, and many more DEGs were included after reanalysis with less stringency (46 DEGs, 32 upregulated and 14 downregulated; Supplementary Fig. 2F).

Downstream KEGG and GO pathway enrichment analysis of the PCR-positive vs. PCR-negative comparison DEGs (*N =* 61) resulted in 1/58 significant KEGG terms and 22/1662 significant GO terms (Table 1). The only KEGG term returned was ‘neuroactive ligand receptor interaction’ (*P*_adj_ = 0.008673; Supplementary Fig. 3A). The top 10 GO terms (by smallest *P*_adj_) were all under the CC category (‘Z disc’, ‘I band’, ‘sarcomere’, ‘contractile fibre part’, ‘myofibril’, ‘contractile fibre’, ‘secretory granule’, ‘rough endoplasmic reticulum’, ‘voltage-gated sodium channel complex’, and ‘focal adhesion’; Supplementary Fig. 3B).

KEGG and GO pathway enrichment analysis of the PCR-positive vs. mock control comparison DEGs (*N =* 46) identified 6/57 significant KEGG terms and 58/1 188 significant GO terms (Table 1). The six enriched KEGG pathways were ‘nicotine addiction’, ‘circadian entrainment’, ‘glutamatergic synapse’, ‘serotonergic synapse’, ‘dopaminergic synapse’, and ‘neuroactive ligand-receptor interaction’ (Supplementary Fig. 3C). The top 10 GO terms ranked by adjusted *P*-value were all in the CC category, including ‘presynaptic membrane’, ‘T-tubule’, ‘excitatory synapse’, ‘cation channel complex’, and ‘ion channel complex’ (Supplementary Fig. 3D).

#### Pooling SYD.1- and SYD.2-infected rats did not yield any significant KEGG or GO terms

To investigate the general host response to infection with rat lungworm, we compared treated (pooled SYD.1- and SYD.2-infected; JS1M_A, JS1U_A, JS2M_A, JS2U_A, JS3M_A, JS3U_A, JS4M_S, JS4U_S, JS5M_S, JS5U_S, JS7M_S, and JS7U_S) rats to control rats (JS6M_M, JS8M_M, JS9M_M, JS6U_M, JS8U_M, and JS9U_M). The individual samples are partially mixed throughout the PCA plot (Supplementary Fig. 4A). The mixing of SYD.1 samples with mock control samples is apparent in the clustered heatmap of the top 200 genes (Supplementary Fig. 4B). Initial differential gene expression analysis of 26 839 transcripts identified no DEGs. A subsequent analysis with relaxed criteria yielded 17 DEGs (12 upregulated, 5 downregulated; Supplementary Fig. 4C). Of the 28 KEGG and 406 GO terms found during downstream functional analysis, none were significant.

#### The basis of the difference between SYD.1- and SYD.2-infected rats relates to vascular smooth muscle contraction

**Figure 4.**
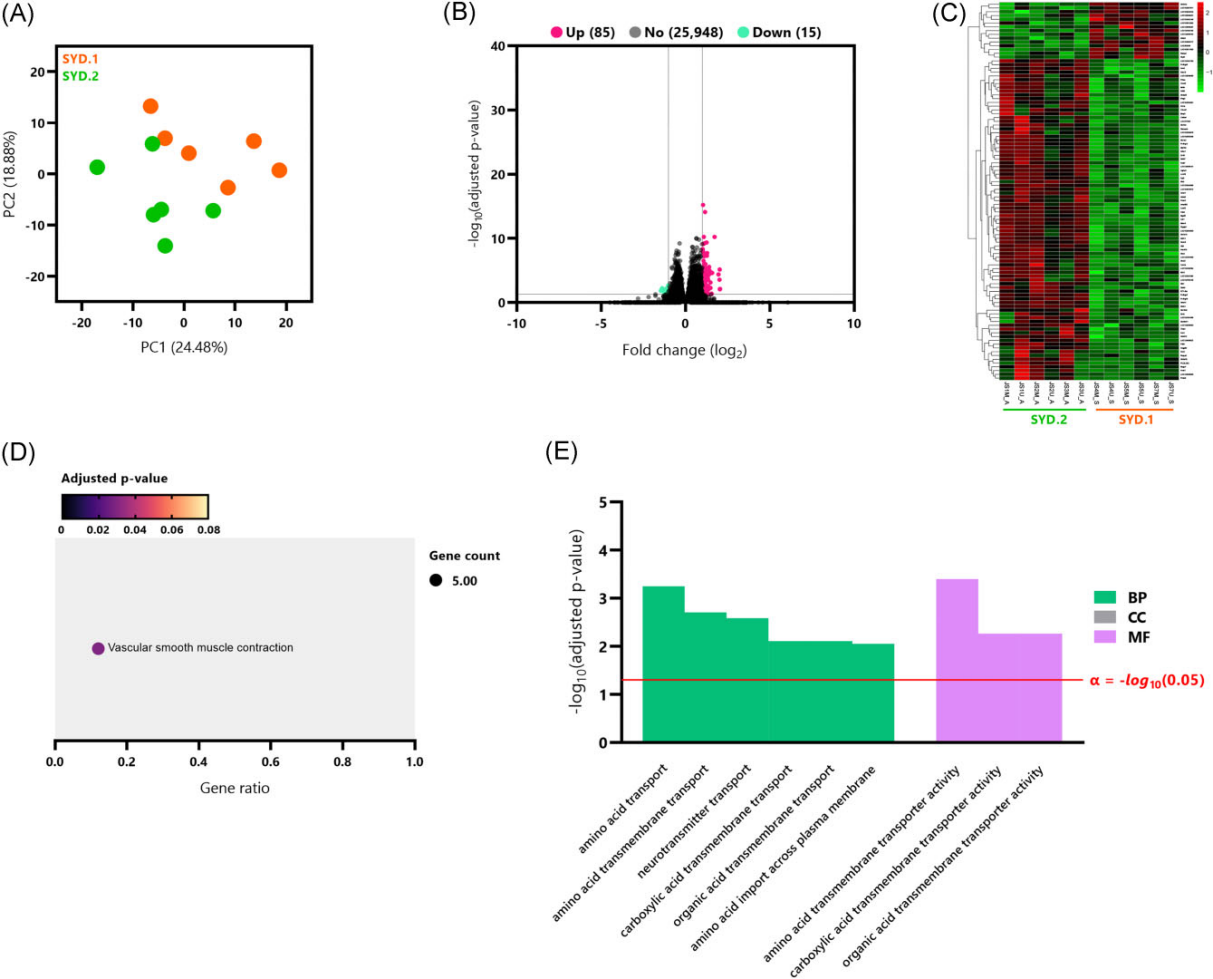
Direct comparison of gene expression profiles between rats infected with *A. cantonensis* isolates SYD.1 and SYD.2 and subsequent DEG functional analyses. (A) PCA plot illustrating sample FKPM distribution for SYD.1 (orange) and SYD.2 (green) groups. (B) Volcano plot depicting DEGs identified by comparing SYD.1 and SYD.2 groups. DEGs were selected based on |log_2_(fold change)| > 1 and *P*_adj_ < 0.05. Upregulated genes are shown in pink, and downregulated in green; all other genes are shown in black. The log_2_(fold change) values are on the *x*-axis, and *P*_adj_ values (transformed by −log_10_) are displayed on the *y*-axis. (C) Hierarchical clustered heatmap of the 100 discovered DEGs between SYD.1 and SYD.2 groups, with gene expression levels [as log_2_(FPKM + 1)] normalized by row (*Z*-scores), which are indicated by a red-green colour gradient. (D) Bubble plot summarizing the significant KEGG (Kyoto Encyclopedia of Genes and Genomes) pathways, with significance determined by *P*_adj_ < 0.05. Bubble size represents the number of DEGs within the pathway, colour indicates the *P*_adj_ value, and the gene ratio (number of DEGs/total genes in the pathway) is shown on the *x*-axis. (E) Bar plot of the top 10 significant GO terms, with significance determined by and *P*_adj_ < 0.05 and top 1-selected by the smallest *P*_adj_. *P*_adj_ values (−log_10_ transformed) are shown on the *y*-axis, and GO terms are on the *x*-axis. Green bars represent biological processes (BP), grey bars represent CC, and pink bars represent MF. The threshold for significance (α = 0.05) is represented by the red line.

To investigate potential differences in host response based on *A. cantonensis* isolate, SYD.1-infected rats (JS4M_S, JS4U_S, JS5M_S, JS5U_S, JS7M_S, and JS7U_S) were compared to SYD.2-infected rats (JS1M_A, JS1U_A, JS2M_A, JS2U_A, JS3M_A, and JS3U_A). The PCA plot demonstrated near-perfect separation between the two groups, indicative of distinct transcriptional profiles (Fig. 4A). Initial differential gene expression analysis of 26 048 transcripts identified four DEGs. A subsequent analysis with relaxed criteria identified 100 DEGs (85 upregulated, 15 downregulated), with the clustered heatmap showing clear separation between the two groups (Fig. 4B and C, respectively). Downstream KEGG and GO enrichment analyses of these 100 DEGs revealed 29/2158 significant KEGG pathways and 1/150 significant GO terms. The sole significant KEGG pathway was ‘vascular smooth muscle contraction’ (Fig. 4D). The top 10 GO terms included seven biological processes related to amino acid transport (‘amino acid transport’, ‘amino acid transmembrane transport’, ‘neurotransmitter transport’, ‘carboxylic acid transmembrane transport’, ‘organic acid transmembrane transport’ and ‘amino acid import across plasma membrane’) and three MF associated with amino acid transmembrane transporter activity (‘amino acid transmembrane transporter activity’, ‘carboxylic acid transmembrane transporter activity’, and ‘organic acid transmembrane transporter activity’; Fig. 4E).

### Adult worm burden differed between SYD.1- and SYD.2-infected rats, but not the quantity of L1s shed in faeces

**Figure 5.**
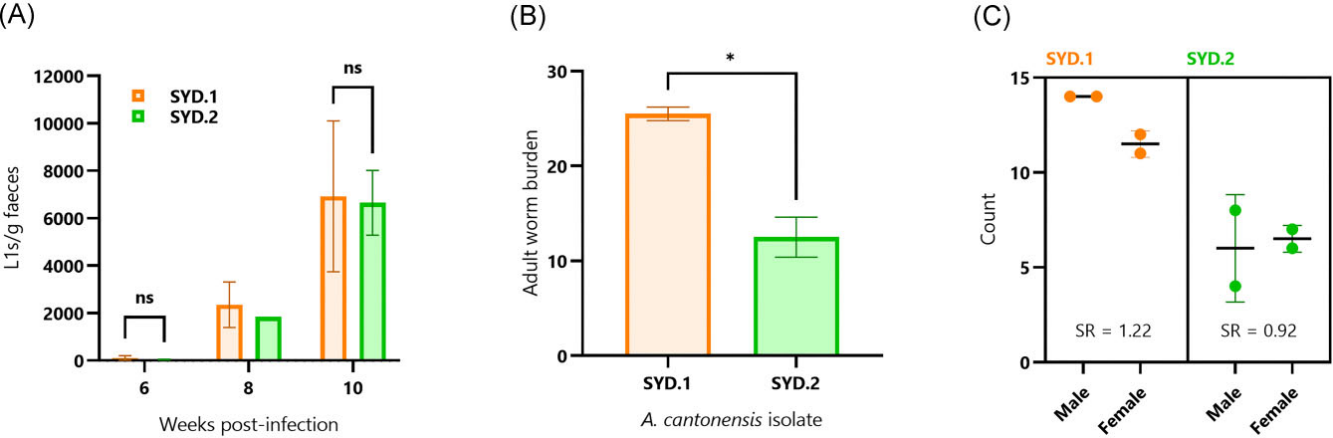
Comparison of L1 shedding and total adult worm burden of rats infected with *A. cantonensis* isolates SYD.1 or SYD.2. (A) Shedding of first-stage larvae (L1s) in faeces of rats infected with *A. cantonensis* isolates SYD.1 or SYD.2. L1s/g faeces of four rats (two infected with SYD.1 and another two infected with SYD.2) were counted at 6-, 8-, and 10-weeks post-infection. Counts were repeated three times, and the average value was taken. Bars indicate the group mean, and error bars show the standard deviation (SD). Note: one sample was excluded (SYD.2, rat 2, week 8) due to equipment failure; hence, error bars are not shown for that group mean. Excluding the week-8 data, the Mann–Whitney test with multiple comparisons confirmed there was no significant difference in L1 shedding between Ac13 and SYD.1 groups at the 6- and 10-week collection points (*P* = .67 and *P* > .99, respectively; indicated by ‘ns’). (B) Mean total adult worm counts from the same cohort of rats infected with SYD.1 and SYD.2 *A. cantonensis* isolates. According to an unpaired *t*-test, the means were significantly different (indicated by the asterisk; *P* < .05). Error bars show the SD. (C) Mean count of male and female adult worms per treatment group, with sex ratios (SR; male:female) shown and SDs indicated by error bars.

A separate cohort of four rats (two per *A. cantonensis* isolate) was monitored for L1 shedding in faecal samples at 6-, 8-, and 10-weeks post-infection. A large increase in average L1s per gram of faeces was observed over time, with a combined average of 74.4 L1s/g at week 6 rising to 2188.5 L1s/g at week 8 and further to 6789.1 L1s/g at week 10. Overall, no discernible difference in L1 shedding over time was observed between rats infected with the SYD.1 or SYD.2 isolates (Fig. 5A). Note that one sample was excluded (SYD.2, rat 2, week 8) due to equipment failure, so error bars (standard deviation; SD) are not shown. Excluding the week-8 data, the Mann–Whitney test with multiple comparisons confirmed there was no significant difference in L1 shedding between SYD.1 and SYD.2 groups at the 6- and 10-week collection points (*P* = .67 and *P* > .99, respectively). The mean total worm count from the SYD.1-infected rats was 25.5 (SD ± 0.7071) and SYD.2-infected rats was 12.5 (SD ± 2.121); the means were significantly different (unpaired *t*-test, *P* = .0145) (Fig. 5B). Sex ratios (male:female) were 1.22 and 0.92 for the SYD.1 and SYD.2 groups, respectively (Fig. 5C).

## Discussion

**Figure 6.**
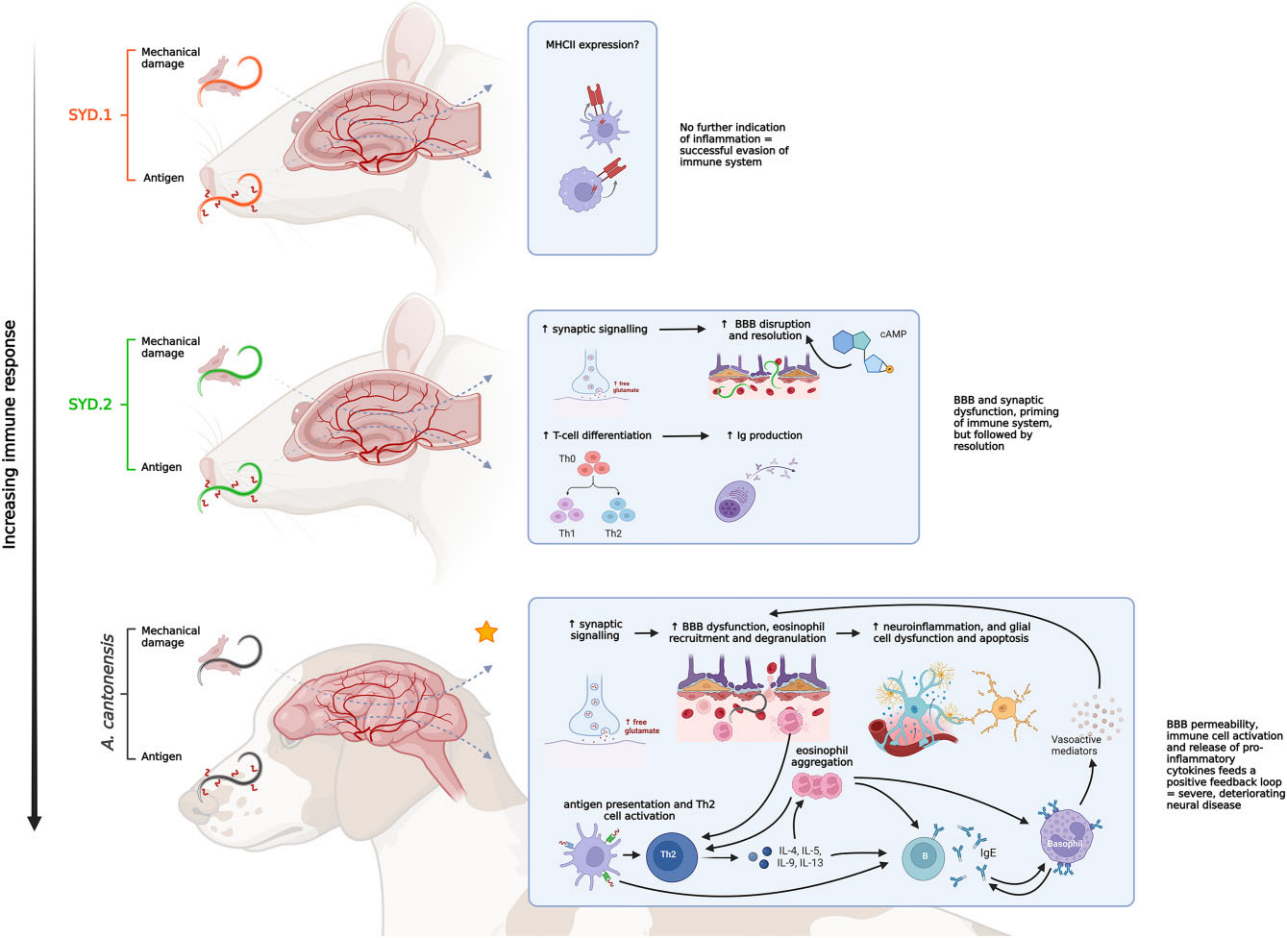
Overview of the host response to infection with *A. cantonensis* in definitive (rat) hosts extrapolated from RNA transcriptomic data from rats infected with the SYD.1 and SYD.2 isolates (*cox*1 haplotype SYD.1 and Ac13, respectively). The response from a non-permissive canine host (indicated with the yellow star) is also shown to highlight the differences between definitive and accidental host responses. The magnitude of the immune response is sorted top to bottom from low to high. The reader is referred to the results and discussion for full details. In brief, this study investigated the transcriptional response in the brain of the *A. cantonensis* definitive (rat) host during early infection with isolates (SYD.1 and SYD.2) from two haplotypes endemic in Australia (SYD.1 and Ac13, respectively). Infection with the SYD.1 isolate resulted in only one DEG—RT1-Db1, which is predicted to enable MHC II receptor activity. Contrastingly, infection with the SYD.2 isolate induced a greater response with 100 DEGs identified. Subsequent functional analyses revealed the enrichment of KEGG pathways such as ‘neuroactive ligand-receptor interaction’, ‘cAMP signalling pathway’, ‘glutamatergic synapse’, ‘intestinal immune network for IgA production’, and ‘Th1 and Th2 cell differentiation’, which is indicative of some innate and cellular immune response activation without progression. In the accidental host, however, the literature supports an over-activation of the immune response, which is ultimately what causes the clinical manifestation of neural angiostrongyliasis. The ability for *A. cantonensis* to evade, supress and/or modulate the immune response of the definitive rat host via excretory-secretory (ES) molecules likely contributes to the apparently low pathogenicity (dose-dependant) in rats when compared to other non-permissive hosts. Created in BioRender (2024) https://BioRender.com/n11o501.

The considerably larger set of DEGs and subsequent enrichment of functional terms in SYD.2-infected rats compared to controls suggests that the SYD.2 isolate (*cox*1 haplotype Ac13) promotes a more pronounced immune response in the CNS than SYD.1 (Fig. 6). The enrichment of inflammatory pathways such as Th1/Th2 cell differentiation and intestinal IgA production indicates adaptive immune actuation, particularly as a ‘type 2’ response dominates during helminth infections in non-permissive hosts (Allen and Maizels 2011, Peng et al. 2012, Yu et al. 2015, Jhan et al. 2021). Dysfunction of glial cells and their synaptic communications has also been observed in models of brain injury (Grewer et al. 2008, Shin and Dixon 2015). Consequently, altered neuronal activity pathways (neuroactive ligand-receptor interaction, cholinergic synapse, and glutamatergic synapse) may reflect direct injury and interruption of synaptic signalling by migrating larvae, additionally supported by their enrichment in rtPCR-positive (‘actively’ infected) rats.

As anticipated, the immune response in the definitive rat host was notably less pronounced than in previous studies using non-permissive murine hosts. Several key inflammatory pathways previously identified in mice were absent in both infected rat groups. For instance, the NF-kB and NOD-like receptor signalling pathways—which regulate numerous inflammatory genes (Capece et al. 2022)—are upregulated in *A. cantonensis*-infected mice (Chen and Wang 2017, Chen et al. 2021, Cheng et al. 2024), but were not detected in our rat study. The NF-kB pathway is one of multiple targets that nematode parasites exploit to modulate host immune responses (Bąska and Norbury 2022). Additionally, expected cytokine signalling pathways (TNF, IL-17, JAK-STAT, MAPK, and TLR), complement, and coagulation cascade activation were not enriched (Lan et al. 2004, Donghui et al. 2024). This muted inflammatory response likely reflects both host co-evolution and parasite immune manipulation through immunomodulating molecules (Okakpu and Dillman 2022).

Notably, SYD.1-infected rats showed higher total worm counts despite exhibiting minimal transcriptional changes compared to controls. This suggests that SYD.1’s enhanced ability to evade host immune detection may contribute to greater parasite survival. Conversely, the more pronounced immune response observed in SYD.2-infected rats may contribute to increased larval killing, resulting in lower worm counts. The fact that L1 shedding was not different between the SYD.1- and SYD.2-infected groups indicates that a crowding effect, or increasing worm burden, may have contributed to a lower larval output in the SYD.1 group (Harris 1982, Kino 1984). However, these interpretations should be considered with caution due to the small sample size (two rats per group) in the worm recovery analysis. Additionally, as these isolates have been maintained in laboratory cycles, their behaviour may not perfectly reflect that of their respective haplotypes in natural settings, where environmental factors and host–parasite interactions may be more complex.

*Angiostrongylus cantonensis* employs various excretory-secretory products (ESPs) for immune evasion, including functional RNAs, proteases, protease-inhibitors, galectins, and antioxidants (Morassutti 2012, Morassutti et al. 2012, Chang et al. 2014). Functional RNAs such as miRNAs produced by *A. cantonensis* L3s and L4s likely modulate the host immune system by altering transcription of immune pathway genes (Li et al. 2014). Metalloproteases secreted by L3s impair pro-cytokine activation and leukocyte migration (Adisakwattana et al. 2012), while protease-inhibitors protect against host proteases and interfere with regulatory pathways (Knox 2007). Galectins prevent mast cell degranulation, inhibit Th1 responses, and suppress innate and humoral responses by acting as ‘antibody sponges’ (Donskow-Łysoniewska et al. 2021). Indeed, *A. cantonensis* galectin-1 induces macrophage apoptosis (Shi et al. 2020) and reduces humoral immune responses in vaccinated mice (Yan et al. 2018). The enrichment of Th1/Th2 cell differentiation pathway in SYD.2-infected rats, together with the enrichment of intestinal IgA production but the absence of other antibody-related pathways, is suggestive of selective immunomodulation by the parasite.

Direct comparison of SYD.1 and SYD.2-infected rats revealed ‘vascular smooth muscle contraction’ as the only significantly enriched KEGG pathway. Pathological damage due to vascular dilation in the brain has been observed in both rats and mice (Jindrák 1970, OuYang et al. 2012, Cheng et al. 2024), potentially resulting from vasoactive mediators released during inflammation, oxidative stress, and impaired endothelial function (Millán Solano et al. 2023). While *A. cantonensis* causes apoptosis of brain astrocytes and microvascular endothelial cells during BBB passage in mice (Guo et al. 2021, Zhou et al. 2022), this appears less pronounced in rats (Zhou et al. 2019). We propose that *A. cantonensis* ESPs may mimic or antagonize endogenous vasoactive mediators such as NO, catecholamines, prostaglandins, and neurotransmitters to facilitate BBB crossing without detection. Neurotransmitters like glutamate and 5-HTP increase following brain injury (Tsuiki et al. 1995, Grewer et al. 2008), which can contribute to BBB hyperpermeability and oedema (Sharma et al. 2019). The identification of ‘glutamatergic synapse’ and ‘serotonergic synapse’ terms in infected rats reflects this BBB dysfunction, while cAMP signalling enrichment in SYD.2-infected rats may indicate attempted resolution of BBB disruption (Tavares et al. 2020) (Fig. 6).

Although both isolates showed similar ‘active’ infection rates (2/6 and 3/6 rtPCR-positive for SYD.1 and SYD.2, respectively), their distinct transcriptional profiles and adult worm burdens support that phenotypic differences exist between *A. cantonensis* haplotypes. The Ac13 haplotype was first detected in Thailand (Rodpai et al. 2016), while the SYD.1 haplotype appears unique to Australia (Mallaiyaraj Mahalingam et al. 2021). These two haplotypes differ by only 1.2% on the partial *cox*1 gene (Rivory et al. 2023), yet isolates of these types experimentally elicited different definitive host transcriptomic responses and abilities to establish infection. This aligns with previous studies showing phenotypic variations between geographical isolates. For instance, Brazilian ac8 and ac9 haplotypes demonstrated different establishment rates and fecundity in rats (Monte et al. 2014). Both haplotypes have been detected in Australian dogs, with the Ac13 haplotype apparently more common (Mallaiyaraj Mahalingam et al. 2021, Baláž et al. 2023). The increased immune response and indication of inflammation in rats infected with the SYD.2 isolate (matching Ac13 *cox*1 haplotype) could potentially account for the observed prevalence in dogs. Specifically, that clinically affected dogs may be more likely to have been infected with the Ac13 haplotype and therefore are more likely to display signs associated with inflammation and BBB dysregulation. Despite the close phylogenetic clustering of Ac13 and SYD.1 (Červená et al. 2019), this study emphasizes the existence of an isolate-dependant response from the host in host–parasite relationships.

This study emphasizes that while molecular tests and the identification of haplotypes are valuable in the surveillance of *A. cantonensis*, the link between virulence or pathogenicity and mitochondrial markers remains only a correlation at this moment. Our study, while providing an approach to establish the link between genotype and phenotype, has limitations. Specifically, the study considered only a single isolate per haplotype. Therefore, confirmation of these results using a larger set of isolates (3–5 recent isolates per haplotype) is warranted. Expanding the genetic analysis to include the nematode genome via population genomics and single nucleotide polymorphism analysis between groups of isolates could potentially identify genes and transcripts under selective pressure, which may be related to the observed phenotype. Additionally, revealing the host response through histopathological or spatial transcriptomics would likely provide a more comprehensive understanding of the host–pathogen interaction, leading to the disease itself and the potential sequelae in the hosts.

## Conclusion

In conclusion, the study highlights significant phenotypic differences between the SYD.1 and SYD.2 isolates of *A. cantonensis*, despite their close genetic similarity. The SYD.2 isolate, belonging to the Ac13 *cox*1 haplotype, induced a more pronounced immune response in rats, with increased inflammation and altered neuronal activity pathways, suggesting a stronger immune activation. Conversely, the SYD.1 isolate appeared to evade host immune detection more effectively, possibly contributing to higher worm burdens. Considering that the inflammatory response to infection observed in non-permissive hosts is the main contributor to disease manifestation, the Ac13 haplotype may be detected in the majority of clinically affected dogs due to Ac13 having a higher propensity to trigger the host’s immune system. It is possible that different haplotypes of *A. cantonensis* pose different disease outcomes in accidentally infected hosts, such as dogs and humans.

## Supplementary Material

ftaf003_Supplemental_Files

## Data Availability

All data used in this study are available at LabArchives (https://dx.doi.org/10.25833/jn6p-3p17). Raw RNA-seq data (fastq files) generated in this study have been deposited in NCBI’s SRA (Sequence Read Archive) and are accessible through the BioProject PRJNA1146323 (https://www.ncbi.nlm.nih.gov/sra/PRJNA1146323).
