# Supplementary material for: Isolate-specific rat brain transcriptional responses to rat lungworm (Angiostrongylus cantonensis)
Source: Pathog Dis. 2025 Feb 19;83:ftaf003. doi: 10.1093/femspd/ftaf003 (PMC11895509; doi:10.1093/femspd/ftaf003)
Supplement: ftaf003_Supplemental_Files [file ftaf003_supplemental_files.zip › Supplementary Materials Legend-Figures.docx]

**Supplementary Figure Legends**

**Supplementary Figure 1** – To assess potential confounding factors within the control group, a differential gene expression analysis was performed comparing uninfected marked and unmarked rats. (A) PCA plot illustrating sample FKPM read distribution based on cage number (colour; black = cage 6, red = cage 9 and grey = cage 9) and marking status (shape; triangle = marked, circle = unmarked). (B) Hierarchical clustered heatmap of the 200 genes between marked and unmarked control rats, sorted by smallest *P*_adj._ Gene expression levels (as *log*_2_(FPKM+1)) were normalised by row (Z-scores) and displayed by the red-green colour gradient. (C) Volcano plot depicting differentially expressed genes (DEGs) identified by comparing marked and unmarked control groups, with DEGs selected based on |*log*_2_(fold change)| > 2. The *log*_2_(fold change) values are on the x-axis, and *P*_adj_ values (transformed by -*log*_10_) are displayed on the y-axis.

**Supplementary Figure 2** – To investigate potential differences in gene expression between AcanR3990-rt(PCR)-positive (i.e. “active” infection) and PCR-negative rats, as well as between PCR-positive and control rats, differential gene expression analyses were performed. Panels A-D compare PCR-positive and PCR-negative rats from both SYD.1 and SYD.2 treatment groups, while panels E-H compare PCR-positive rats to control rats. (A) PCA plot illustrating sample distribution for PCR-positive (black outline) and PCR-negative rats, with treatment group indicated by colour (SYD.1 = orange, SYD.2 = green). (B) Hierarchical clustered heatmap of the top 200 differentially expressed genes (sorted by smallest *P*_adj_) between PCR-positive and PCR-negative rats. Gene expression levels (as *log*_2_(FPKM+1)) were normalised by row (Z-scores), which are indicated by red-green colour. (C) Volcano plot depicting differentially expressed genes (DEGs) identified by comparing PCR-positive and PCR-negative rats, with upregulated genes in pink, downregulated genes in green, and non-significant genes in black. DEGs were selected based on |*log*_2_(fold change)| > 1 and *P*_adj_ < 0.05. The *log*_2_(fold change) values are on the x-axis, and *P*_adj_ values (transformed by -*log*_10_) are displayed on the y-axis. (D) Hierarchical clustered heatmap of the 61 discovered DEGs between PCR-positive and PCR-negative rats. Gene expression levels (as *log*_2_(FPKM+1)) were normalised by row (Z-scores), which are indicated by red-green colour. (E) PCA plot illustrating sample distribution for PCR-positive (black outline) and control rats (blue), with treatment group (SYD.1 and Ac13) indicated by color (orange and green, respectively). (F) Hierarchical clustered heatmap of the top 200 differentially expressed genes (chosen according to smallest *P*_adj_ values) between PCR-positive and mock control rats. Gene expression levels (as *log*_2_(FPKM+1)) were normalised by row (Z-scores; red-green colour). (G) Volcano plot depicting differentially expressed genes (DEGs) identified by comparing PCR-positive and control rats, with upregulated genes in pink, downregulated genes in green, and all other genes in black. DEGs were selected based on |*log*_2_(fold change)| > 1 and *P*_adj_ < 0.05. The *log*_2_(fold change) values are on the x-axis, and *P*_adj_ values (transformed by -*log*_10_) are displayed on the y-axis. (H) Hierarchical clustered heatmap of the 46 discovered DEGs between PCR-positive and mock control rats. Gene expression levels (as *log*_2_(FPKM+1)) were normalised by row (Z-scores), which are indicated by red-green colour.

**Supplementary Figure 3** – Follow-up functional analyses of detected differentially expressed genes (DEGs) between AcanR3990-rt(PCR)-positive (i.e., detectable or “active” infection) and PCR-negative rats (panels A and B), as well as between PCR-positive and control rats (panels C and D). Functional enrichment analysis included KEGG (Kyoto Encyclopedia of Genes and Genomes) pathway enrichment (panels A and C), and GO (Gene Ontology) term enrichment (panels B and D). (A) Bubble plot depicting the significant KEGG pathways identified in the PCR-positive versus PCR-negative comparison. Significance was determined by *P*_adj_ < 0.05. Bubble size represents the number of DEGs within the pathway, colour indicates the *P*_adj_, value, and the gene ratio (number of DEGs / total genes in the pathway) is displayed on the x-axis. (B) Bar plot of the top 10 significant GO terms (sorted by smallest *P*_adj_) identified in the PCR-positive *vs*. PCR-negative comparison. Significance was determined by *P*_adj_ < 0.05. Transformed *P*_adj_ values (by -*log*_10_) are shown on the y-axis, and GO terms are on the x-axis. Green bars represent Biological Processes (BP), grey bars represent Cellular Components (CC), and pink bars represent Molecular Functions (MF). (C) Bubble plot depicting the significant KEGG pathways identified in the PCR-positive *vs*. control comparison. (D) Bar plot of the top 10 significant GO terms identified in the PCR-positive *vs*. control comparison. Methodology for panels C and D is identical to panels A and B, respectively.

**Supplementary Figure 4** – Results from differential gene expression analysis performed to compare transcriptomes of infected rats (*A. cantonensis* SYD.1- or SYD.2-infected) to control rats. (A) PCA plot illustrating sample FKPM read distribution for treated rats (circles; SYD.1 = orange, SYD.2 = green) and control rats (diamonds; blue). (B) Hierarchical clustered heatmap of the top 200 genes sorted by smallest adjusted p-value (*P*_adj_) between infected and mock control groups. Gene expression levels (as *log*_2_(FPKM+1)) were normalised by row (Z-scores; shown in red-green colours). (C) Volcano plot depicting differentially expressed genes (DEGs) identified by comparing treated and control groups. DEGs were selected by |*log*_2_(fold change)| > 1 and *P*_adj_ < 0.05. The *log*_2_(fold change) values are on the x-axis, and *P*_adj_ (transformed by -*log*_10_) are displayed on the y-axis.
