# Supplementary material for: Isolate-specific rat brain transcriptional responses to rat lungworm (Angiostrongylus cantonensis)
Source: Pathog Dis. 2025 Feb 19;83:ftaf003. doi: 10.1093/femspd/ftaf003 (PMC11895509; doi:10.1093/femspd/ftaf003)
Supplement: ftaf003_Supplemental_Files [file ftaf003_supplemental_files.zip › SuppTables_FINAL.docx]

**Supplementary Table 1** – Experimental design summary, with RNA sequencing and mapping statistics for each sample

| Sample name | Marked? |  | Cage # | Treatment group | # Raw reads | Raw bases (G) | Q20 | Q30 | GC% | # Mapped reads (%) | # Unique mapped reads (%) |
| --- | --- | --- | --- | --- | --- | --- | --- | --- | --- | --- | --- |
| JS1M_A | Y |  | 1 | SYD.2  (Ac13 haplotype) | 64087286 | 9.61 | 98.23 | 95.06 | 47.51 | 60847368 (97.39%) | 59369022 (95.03%) |
| JS1U_A | N |  |  |  | 69357064 | 10.4 | 98.23 | 95.12 | 47.47 | 66104962 (97.37%) | 64430561 (94.91%) |
| JS2M_A | Y |  | 2 |  | 68447182 | 10.27 | 98.12 | 94.9 | 47.31 | 64634993 (97.09%) | 63012573 (94.65%) |
| JS2U_A | N |  |  |  | 63179318 | 9.48 | 98.06 | 94.8 | 47.18 | 60104648 (97.04%) | 58442094 (94.35%) |
| JS3M_A | Y |  | 3 |  | 59984030 | 9 | 98.22 | 95.14 | 46.56 | 56229547 (97.09%) | 54795709 (94.62%) |
| JS3U_A | N |  |  |  | 63992466 | 9.6 | 98.15 | 94.93 | 47.73 | 60533144 (97.24%) | 58957501 (94.7%) |
| JS6M_M | Y |  | 6 | Control | 65159194 | 9.77 | 98.05 | 94.78 | 46.39 | 61716394 (96.97%) | 60026587 (94.32%) |
| JS6U_M | N |  |  |  | 65730830 | 9.86 | 98.16 | 94.99 | 46.52 | 62572190 (97.1%) | 60974825 (94.63%) |
| JS8M_M | Y |  | 8 |  | 64488754 | 9.67 | 98.11 | 94.9 | 45.28 | 60399382 (96.92%) | 58955291 (94.61%) |
| JS8U_M | N |  |  |  | 65448626 | 9.82 | 98.15 | 94.86 | 45.91 | 62661905 (97.22%) | 61140127 (94.86%) |
| JS9M_M | Y |  | 9 |  | 66849932 | 10.03 | 98.08 | 94.76 | 44.66 | 63503802 (96.9%) | 61953186 (94.54%) |
| JS9U_M | N |  |  |  | 65204790 | 9.78 | 98.06 | 94.69 | 44.72 | 62133949 (96.78%) | 60567071 (94.34%) |
| JS4M_S | Y |  | 4 | SYD.1 | 1.03E+08 | 15.46 | 97.89 | 94.31 | 45.32 | 97025565 (96.82%) | 94800792 (94.6%) |
| JS4U_S | N |  |  |  | 64201306 | 9.63 | 98.17 | 95.01 | 46.22 | 60810241 (97.02%) | 59349831 (94.69%) |
| JS5M_S | Y |  | 5 |  | 61365902 | 9.2 | 98.03 | 94.79 | 47.08 | 57972621 (96.87%) | 54909104 (91.75%) |
| JS5U_S | N |  |  |  | 62521988 | 9.38 | 98.03 | 94.77 | 45.85 | 58332618 (96.81%) | 56843832 (94.34%) |
| JS7M_S | Y |  | 7 |  | 62433872 | 9.37 | 98.14 | 94.98 | 46.27 | 58637748 (97.03%) | 57160607 (94.58%) |
| JS7U_S | N |  |  |  | 66394344 | 9.96 | 98.15 | 94.92 | 46.6 | 63233402 (97.17%) | 61574411 (94.62%) |
| Average  ±SD | | | | | 66769271.33 ±9073296.94 | 10.02 ±1.36 | 98.11 ±0.08 | 94.87 ±0.18 | 46.37 ±0.91 | 97.05 ±0.18 | 94.45 ±0.68 |

G = Gigabyte; Y = Yes; N = No; Q20 = Quality score 20 (probability of incorrect base call = 1/100); Q30 = Quality score 30 (probability of incorrect base call = 1/1000); GC% = percentage of Guanine-Cytosine content; SD = standard deviation (population).

**Supplementary Table 2** – Ct-values from real-time (rt) partial Beta (ß)-actin and AcanR3990 (*Angiostrongylus*) PCR on brain tissue aliquots

|  |  | ß-actin rtPCR | | | AcanR3990 rtPCR | | |
| --- | --- | --- | --- | --- | --- | --- | --- |
| Sample name | **Treatment group** | **Aliquot 1** | **Aliquot 2** | **Result* (PASS/FAIL)** | **Aliquot 1** | **Aliquot 2** | **Result** (+/-)** |
| JS1M_A | SYD.2 (Ac13 haplotype) | 21.05438721 | 21.7338361 | PASS | 20.83 | 21.72 | **­+** |
| JS1U_A |  | 20.56052819 | 22.14782399 | PASS |  |  | **-** |
| JS2M_A |  | 20.98545861 | 21.06448489 | PASS |  |  | **-** |
| JS2U_A |  | 20.66335031 | 22.18645594 | PASS |  |  | **-** |
| JS3M_A |  | 20.7037385 | 22.22090827 | PASS | 25.58 | 24.40 | **+** |
| JS3U_A |  | 21.15039746 | 21.53965482 | PASS | 23.13 | 28.08 | **+** |
| JS6M_M | Control | 20.97562879 | 21.1016863 | PASS |  |  | **-** |
| JS6U_M |  | 20.94980667 | 21.23308619 | PASS |  |  | **-** |
| JS8M_M |  | 20.67857512 | 21.05587908 | PASS |  | 39.54 | **-** |
| JS8U_M |  | 20.76230504 | 21.30856409 | PASS |  | 39.19 | **-** |
| JS9M_M |  | 20.42470364 | 20.99281319 | PASS |  |  | **-** |
| JS9U_M |  | 20.3822534 | 21.08692803 | PASS |  | 39.56 | **-** |
| JS4M_S | SYD.1 | 21.03446998 | 22.02573806 | PASS |  | 39.69 | **-** |
| JS4U_S |  | 20.53799345 | 21.4935261 | PASS |  | 31.06 | **+** |
| JS5M_S |  | 20.59890398 | 22.52715069 | PASS |  |  | **-** |
| JS5U_S |  | 20.66488043 | 21.71573168 | PASS |  | 38.64 | **-** |
| JS7M_S |  | 20.72177702 | 21.16995749 | PASS |  | 38.72 |  |
| JS7U_S |  | 20.94973746 | 21.37863453 | PASS |  | 20.14 | **+** |

*Both Ct-values < 30 = PASS; all other combinations = FAIL.
**At least one Ct-value < 35 = positive (+); all other combinations = negative (-).
