# Supplementary figures and images for: Isolate-specific rat brain transcriptional responses to rat lungworm (Angiostrongylus cantonensis)
Source: Pathog Dis. 2025 Feb 19;83:ftaf003. doi: 10.1093/femspd/ftaf003 (PMC11895509; doi:10.1093/femspd/ftaf003)

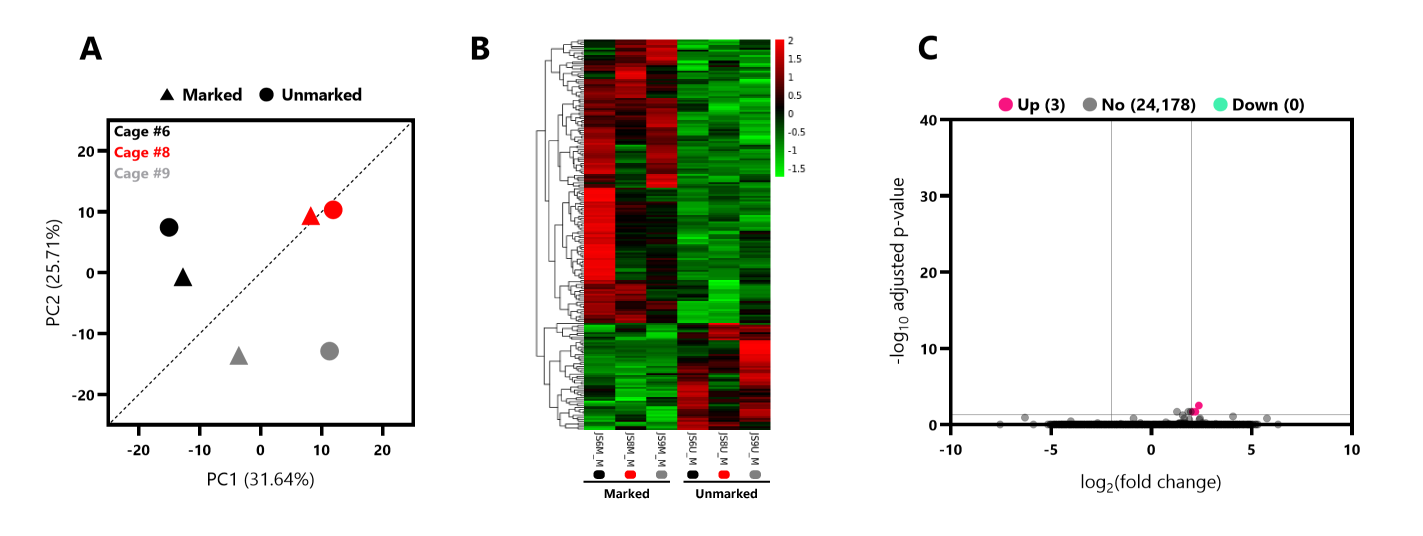

Supplement: ftaf003_Supplemental_Files [file ftaf003_supplemental_files.zip › Supplementary figure 1.tif]

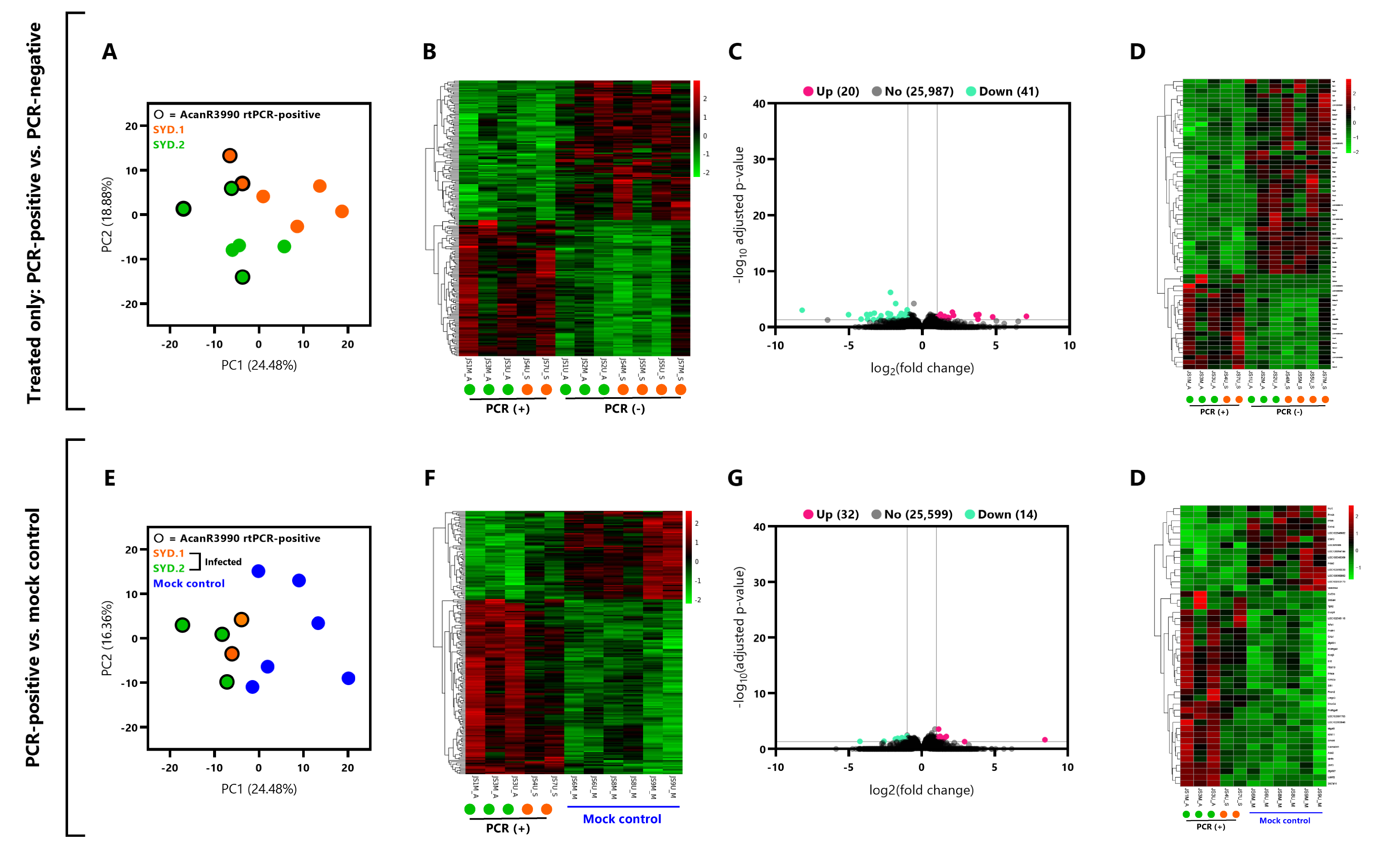

Supplement: ftaf003_Supplemental_Files [file ftaf003_supplemental_files.zip › Supplementary figure 2.tif]

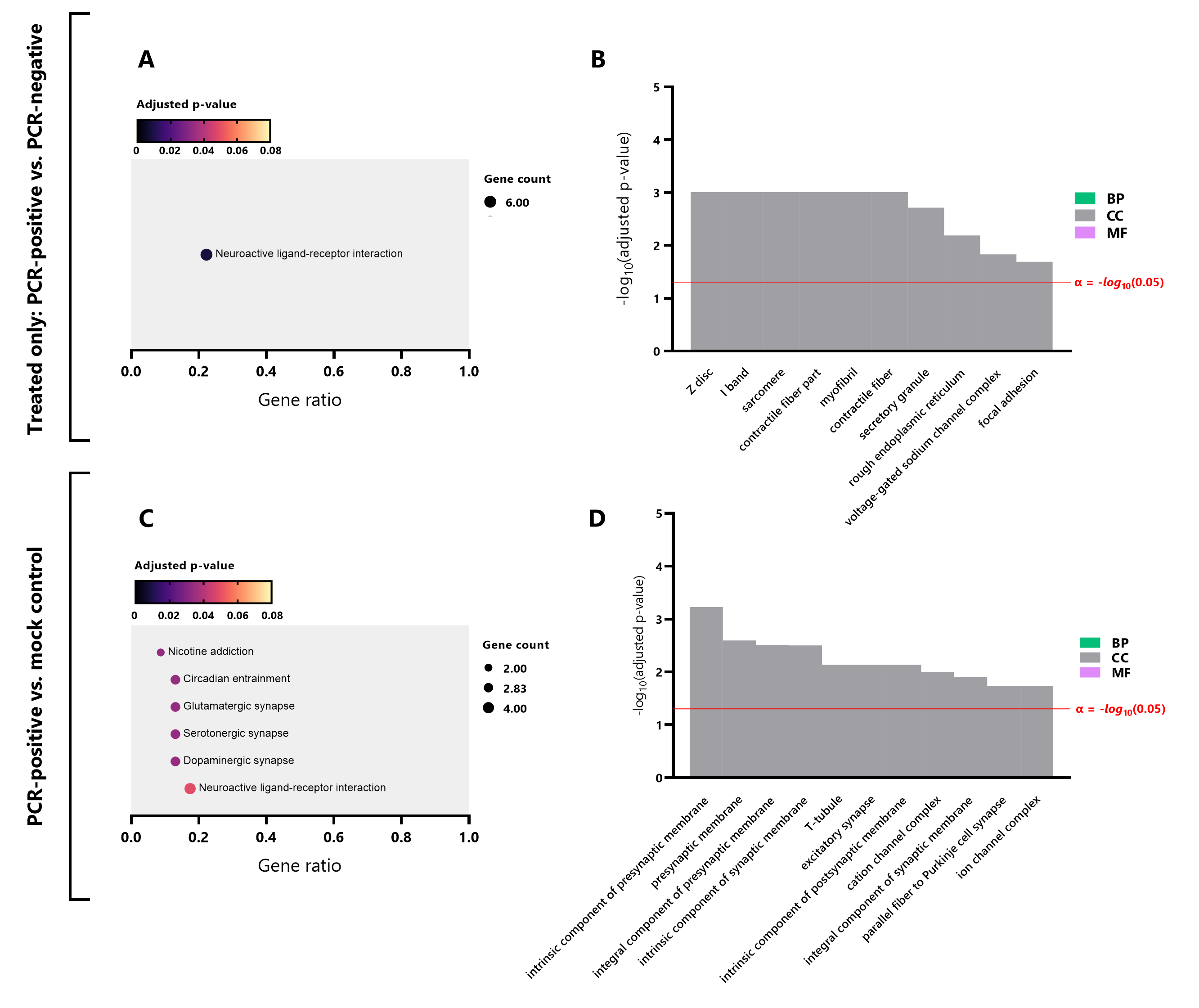

Supplement: ftaf003_Supplemental_Files [file ftaf003_supplemental_files.zip › Supplementary figure 3.tif]

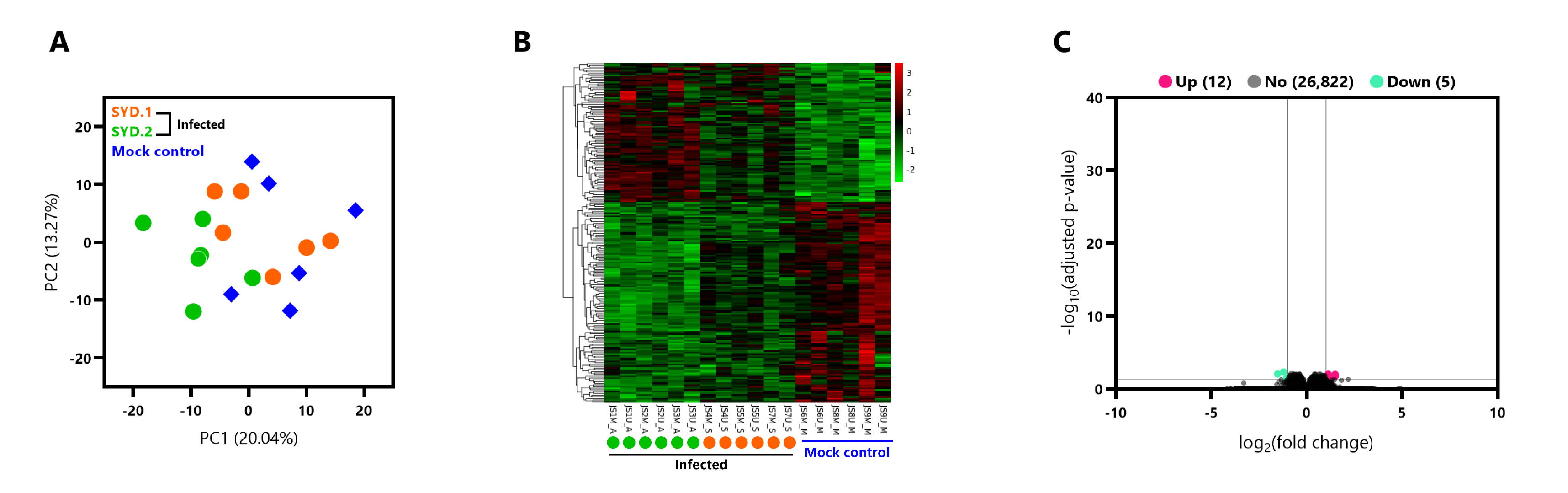

Supplement: ftaf003_Supplemental_Files [file ftaf003_supplemental_files.zip › Supplementary figure 4.tif]
